# Local Drug Delivery Systems as Novel Approach for Controlling NETosis in Periodontitis

**DOI:** 10.3390/pharmaceutics16091175

**Published:** 2024-09-06

**Authors:** Adina Bianca Boșca, Elena Dinte, Carmen Mihaela Mihu, Alina Elena Pârvu, Carmen Stanca Melincovici, Alina Simona Șovrea, Mariana Mărginean, Anne-Marie Constantin, Anida-Maria Băbțan, Alexandrina Muntean, Aranka Ilea

**Affiliations:** 1 Department of Histology, Faculty of Medicine, “Iuliu Hațieganu” University of Medicine and Pharmacy, 400012 Cluj-Napoca, Romania; bianca.bosca@umfcluj.ro (A.B.B.);; 2 Department of Pharmaceutical Technology and Biopharmaceutics, Faculty of Pharmacy, “Iuliu Hațieganu” University of Medicine and Pharmacy, 400012 Cluj-Napoca, Romania; 3 Department of Pathophysiology, Faculty of Medicine, “Iuliu Hațieganu” University of Medicine and Pharmacy, 400012 Cluj-Napoca, Romania; 4 Department of Oral Rehabilitation, Faculty of Dentistry, “Iuliu Hațieganu” University of Medicine and Pharmacy, 400012 Cluj-Napoca, Romaniaaranka.ilea@umfcluj.ro (A.I.); 5 Department of Paediatric Dentistry, Faculty of Dentistry, “Iuliu Hațieganu” University of Medicine and Pharmacy, 400012 Cluj-Napoca, Romania

**Keywords:** NETosis, polymorphonuclear neutrophils, periodontitis, local drug delivery systems, diagnostic biomarkers

## Abstract

Periodontitis is a chronic inflammation caused by periodontopathogenic bacteria in the dental biofilm, and also involves the inflammatory-immune response of the host. Polymorphonuclear neutrophils (PMNs) play essential roles in bacterial clearance by multiple mechanisms, including the formation of neutrophil extracellular traps (NETs) that retain and destroy pathogens. During PD progression, the interaction between PMNs, NETs, and bacteria leads to an exaggerated immune response and a prolonged inflammatory state. As a lesion matures, PMNs accumulate in the periodontal tissues and die via NETosis, ultimately resulting in tissue injury. A better understanding of the role of NETs, the associated molecules, and the pathogenic pathways of NET formation in periodontitis, could provide markers of NETosis as reliable diagnostic and prognostic tools. Moreover, an assessment of NET biomarker levels in biofluids, particularly in saliva or gingival crevicular fluid, could be useful for monitoring periodontitis progression and treatment efficacy. Preventing excessive NET accumulation in periodontal tissues, by both controlling NETs’ formation and their appropriate removal, could be a key for further development of more efficient therapeutic approaches. In periodontal therapy, local drug delivery (LDD) systems are more targeted, enhancing the bioavailability of active pharmacological agents in the periodontal pocket and surrounding tissues for prolonged time to ensure an optimal therapeutic outcome.

## 1. Introduction

Periodontitis (PD) is the sixth most frequent disease worldwide, affecting 20 to 50% of the global population [1], with severe forms in 11.2% of adults [2]. This chronic inflammatory disease has important consequences on the general health due to its increased systemic inflammatory burden, which is the main link between PD and cardiovascular diseases, diabetes mellitus, rheumatoid arthritis (RA), and metabolic syndrome [3,4,5,6,7].

Periodontal pathogens emerging from the dysbiosis of bacterial species present in the dental biofilm trigger and maintain the local chronic non-resolving inflammatory-immune response of the host [8,9]. In PD patients, the imbalance between defense mechanisms and pathogens’ overgrowth leads to chronic inflammation, periodontal tissue destruction, pocket formation, alveolar bone resorption, and finally tooth loss [9,10].

The common denominator in the inter-relation between PD and systemic diseases is the inflammatory-immune reaction orchestrated by polymorphonuclear neutrophils (PMNs), the main players fighting against microbial challenge [5,11].

In susceptible individuals, inefficient resolution of inflammation leads to prolonged activation of local PMNs, which become dysfunctional and inefficient for bacterial clearance within periodontal tissues, whereas systemic PMNs become hyperreactive and participate in oxidative stress, metainflammation, and autoimmunity; these are complex mechanisms with a significant impact on general health status [8,10,12]. Therefore, there is an urgent need to develop effective therapies and preventive approaches by addressing the complex pathogenic mechanisms, targeting both the microbial-derived factors and the aberrant immune-inflammatory response of the host [13].

This narrative review aimed to present the pathogenic mechanisms of NETosis and, particularly, their involvement in the pathogenesis of periodontitis. Furthermore, various novel therapeutic approaches are discussed, focusing on the advantages of local drug delivery (LDD) systems used as an adjunctive therapy to control NETosis-related periodontal tissue destructions and to optimize the outcome of conventional periodontal treatment.

## 2. Pathogenic Mechanisms Involving PMNs and NET Formation

PMNs are essentially involved in the inflammatory-immune response against invading pathogens, and create an antibacterial environment through multiple mechanisms: secreting reactive oxygen species (ROS), releasing proteases and toxic enzymes via degranulation, killing pathogens in phagosomes via phagocytosis, and the recently described mechanism of trapping pathogens by formation of neutrophil extracellular traps (NETs) [14]. Genetic regulation leading to the secretion of pro-inflammatory cytokines is generally dependent on nuclear factor-kappa B (NF-κB), which can be activated by the pattern-recognition receptors (PRR) or by oxidation [11,15]. Furthermore, PMNs have phagocytose cellular debris [8,10] and have protective functions by secreting anti-inflammatory resolvins and sequestering pro-inflammatory factors [11,15].

ROS are used to alter and inhibit the activity of other molecules for signal transmission and antimicrobial effect performed both intra- and extracellularly [16,17]. Oxidative stress results from the imbalance between the generation of ROS and the ability of biological systems to neutralize these reactive products. Oxidative stress alters cellular structures: lipid peroxidation damages cell membranes and lipoproteins, protein oxidation causes loss of or impaired enzymatic activity, deoxyribonucleic acid (DNA) oxidation causes mutagenicity and genotoxicity, and free carbohydrate oxidation generates reactive carbonyls, non-enzymic glycosylation, and the formation of advanced glycation end products (AGEs) [18]. Besides ROS, the other important reactive species are the reactive nitrogen species (RNS), comprising nitric oxide (NO·) and the reactive radical peroxynitrite anion (ONOO−) [18], which affect mitochondrial functions and trigger cell death [19]. Although inflammation induces oxidant injury, the reverse sequence of events is also true: when produced excessively and for prolonged periods, ROS can promote oxidative stress and associated chronic inflammation. Thus, inflammation and oxidative stress are interrelated [11,17].

NETs represent part of PMNs’ antimicrobial arsenal, but also play essential roles in autoimmunity and various pathophysiological conditions [20,21]. Numerous studies have demonstrated that NETs also represent a link between inflammatory, non-specific immunity, and acquired, specific immunity, by their interaction with other leukocytes [22,23]. NETs are capable of modulating the inflammatory and immune responses by activating T helper (CD4) and cytotoxic (CD8) lymphocytes [23], stimulating macrophages to secrete interleukins 1β and 18 (IL-1β and IL-18) during activation of NLRP3 inflammasome [24,25], and promoting the release of other cytokines, including tumor necrosis factor-α (TNF-α), IL-6 and IL-8 [26,27].

NET formation can be induced by multiple triggers, including microorganisms or pathogenic factors (e.g., lipopolysaccharides—LPS) and through different mechanisms that could be inter-related [22]. NET release can also be triggered by “sterile”, endogenous stimuli, including immune complexes, damage-associated molecular patterns (DAMPs), and inflammatory mediators released at the site of the lesion [10,20]. NET formation is controlled by complex signaling pathways involving various receptors, such as: toll-like receptors (TLRs), immunoglobulin G—IgG-Fc receptors, signal inhibitory receptors on leukocytes-1(SIRL-1), and receptors for cytokines and for AGEs (RAGEs) [28,29].

NETs are in the form of web-like strands secreted by activated PMNs, composed of extruded de-condensed nuclear or mitochondrial DNA associated with antimicrobial peptides (AMPs) such as histones, human neutrophil elastase (NE), myeloperoxidase (MPO), and others. Interestingly, the peptide composition of NETs may vary, depending on the stimulus triggering NETosis [30]. NETs released outside PMNs provide high concentrations of AMPs in the extracellular environment, thus enhancing the bactericidal activity [22,31,32,33,34,35,36]. Other cellular components also play important roles in NET formation: calcium ions released from the endoplasmic reticulum initiate the cascade leading to ROS formation, whereas actin microfilaments and microtubules enable the re-arrangement of the cytoskeleton during NET formation [37,38,39].

Various components of NETs play essential roles in immobilizing pathogens to prevent local and systemic dissemination, destroying microbes and degrading toxic substances derived from bacteria [40]. The antimicrobial function of NETs occurs mainly due to the high accumulation of AMPs at the site of immobilized pathogens [14]. Additionally, citrullinated histones in the NETs could have antimicrobial activity, independently of the AMPs, by damaging the bacterial cell wall [41].

**Figure 1:**
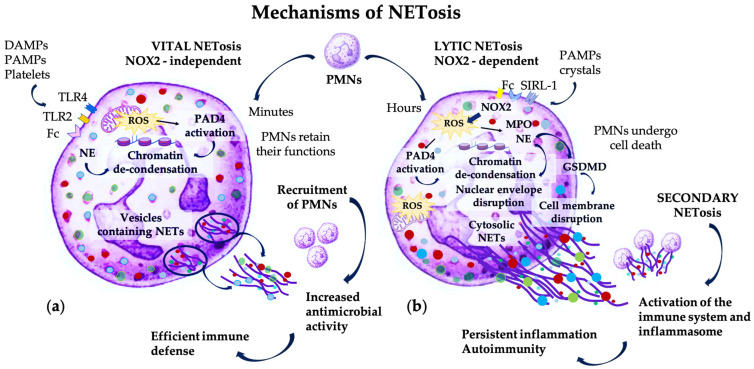
The two mechanisms of NETosis induced by the interaction of various stimuli, such as DAMPs and PAMPs, with surface receptors on PMNs: TLRs, Fc fraction of complement, or SIRL-1. (**a**) Vital, non-lytic NETosis is NOX2-independent and occurs upon short- and low-intensity stimulation; mitochondrial ROS activate PAD4, which citrullinates histones and induces chromatin de-condensation; NE is translocated in the nucleus and participates in chromatin de-condensation; DNA and AMP complexes are transported by vesicles and released extracellularly; PMNs retain their viability and functions, and also attract new PMNs for increased and efficient antimicrobial defense; (**b**) suicidal, lytic NETosis is NOX2-dependent and occurs upon chronic and intense stimulation; NOX2 induces formation of ROS which activate PAD4 and enable the release of MPO and NE from the granules; NE and MPO are translocated in the nucleus, participating in chromatin de-condensation and disruption of the nuclear envelope; DNA–AMP complexes formed in the cytosol are extruded by the disruption of the cell membrane due to activation of GSDMD; PMNs undergo cell death and accumulation of NETs induces secondary NETosis in newly recruited PMNs, with subsequent activation of the immune system and inflammasome due to presence of molecules released by pathogens—PAMPs, and substances derived from tissue damage, like DAMPs and ROS—resulting in persistent inflammation and autoimmunity.

Two main mechanisms of NET formation are recognized, with complex sequential pathways, depending on the stimuli activating the PMNs and resulting in the different composition, activity, and evolution of NETs [10,42,43,44] (Figure 1).

Mechanistically, NETs’ release is modulated by several proteins, but is mainly dependent on ROS formed by nicotinamide adenine dinucleotide phosphate (NADPH)-oxidase (NOX2) [45]. This is the classical pathway of NET formation, involving the disruption of the nuclear envelope and cell membrane, followed by the extrusion of nuclear DNA, and eventually leading to NETosis, a particular type of programmed active cellular death [6,28]. However, alternative NOX2-independent pathways have been described, and these mechanisms are regulated by TLR signaling, involve calcium-dependent mitochondrial ROS formation, and NETs are extruded by vesicular transport, preserving PMNs’ integrity [15,22,46].

Shorter and less intense stimulation of PMNs results in “vital” or “non-lytic” NETosis, in response to bacteria, pathogen-associated molecular patterns (PAMPs), activated platelets, or complement components for rapid antibacterial effect. This pathway is NOX2-independent, and involves the interaction between TLR4 and high mobility group box 1 (HMGB1), resulting from platelets and damaged cells [47]. HMGB1 is an alarmin capable of inhibiting the clearance of apoptotic cells, and leading to persistent inflammation [48]. Some authors have shown that this NOX2-independent pathway is based on mitochondria-derived ROS, leading to the activation of peptidyl arginine deiminase-4 (PAD4), which citrullinates histone H3 and promotes chromatin de-condensation [22,43,49,50,51]. DNA associated with granular proteins is released extracellularly by vesicular transport, without causing cell membrane disruption. Thus, PMNs remain viable and retain their chemotactic and phagocytic activities. Moreover, NETs formed by vital NETosis attract additional PMNs to enhance antimicrobial defense [15,32,52,53]. NETs have been reported as forming within 15 min after PMN stimulation with LPS, for a faster antibacterial effect [32,44]. Some studies have reported that, in non-lytic NETosis, NETs may consist of extruded mitochondrial DNA. However, it is still unclear if the amount of mitochondrial DNA is sufficient for effective NET formation, given the reduced number of mitochondria in PMNs [54,55]. Since PMNs remain viable, they preserve their ability to destroy phagocytosed bacteria. Notably, vital NET formation seems to be the main mechanism involved in infections because it enables the coexistence of both NET release and conventional PMN functions [56]. A particular form of NOX2-independent “vital” NETosis was observed by Pilsczek et al. in response to severe infection with *Staphylococcus aureus*. This mechanism was characterized by rapid NET formation, up to one hour, through the vesicular transport of nuclear chromatin extracellularly. Proteolytic activity of NETs was limited, but, since nuclear and plasma membranes were not disrupted, PMNs’ viability and normal functions were preserved. However, through the subsequent rupture of the nuclear envelope and DNA release in the cytosol, this mechanism could lead to cell death [52].

By contrast, chronic and intense stimulation results in the formation of NETs composed of nuclear DNA as consequence of “lytic” or “suicidal” NETosis [14,28], which is prone to induce autoimmunity and collateral tissue breakdown [57]. The recognition of pathogens or sterile stimuli by PMN receptors triggers the release of intracellular calcium ions from the endoplasmic reticulum, which activate Protein kinase C (PKC) and NOX complex [42]. The activation of NOX2 occurs through phosphorylation by PKC and with the contribution of the Raf-MEK-ERK signaling pathway. NOX2 induces the formation of ROS: superoxide, hydrogen peroxide, and hypochlorous acid. NO donors can promote NET formation through a pathway also involving ROS [58]. ROS upregulate PAD4 responsible for histone citrullination and chromatin de-condensation. Furthermore, ROS dissociate azurophilic granules, releasing the enzymes into the cytoplasm. MPO produces hypochlorite anions to activate NE, a serine protease, which breaks down the cytoskeleton. MPO and NE translocate into the nucleus and degrade histones, contributing to the de-condensation of nuclear chromatin and the disruption of the nuclear envelope [33,34,44,59]. In the cytoplasm, chromatin combines with enzymes released from the cytosolic granules, such as cathelicidin, AMPs, NE, MPO, and proteinase-3 (PR3), to form DNA–AMPs complexes. Later, NE activates gasdermin D (GSDMD), a protein responsible for disrupting the cell membrane and causing the formation of pores through which the DNA–AMP complexes are extruded in the extracellular environment [60]. “Suicidal” NETosis lasts for two to four hours and results in a prolonged antimicrobial effect, beyond PMNs’ lifespan [10,43,44,57].

Excessive NET formation and NETosis underlie numerous autoimmune diseases, including: RA, systemic lupus erythematosus (SLE), preeclampsia, vasculitis, and coagulopathies [39].

In vitro studies performed by Pieterse et al. demonstrated that NET release was selective in response to different types of LPS, and NETosis could be “vital” or “suicidal”, depending on culture conditions. Thus, in a serum-free and platelet-free medium, “suicidal” NETosis occurred in a ROS-dependent manner and involved autophagy. Conversely, in the presence of platelets, NET formation was immediate, and PMNs remained viable. Platelets can activate PMNs by the interaction between TLR4 and P-selectin (CD62P), without ROS formation or autophagy [61]. Simultaneous activation of PMNs and platelets seems to be responsible for the amplified response against pathogenic bacteria [62].

Several studies demonstrated the NET-associated NET formation, which is activated in newly arrived PMNs at the site of inflammation [22,24,63,64]. In theory, the removal of excessive NETs could prevent this feed-forward mechanism. However, Agarwal et al. reported that mechanical disruption or enzymatic degradation of NETs by deoxyribonuclease I (DNase I) is the trigger for new PMN activation and further NET formation—a mechanism called “secondary NETosis” [63].

Both the timing and the magnitude of the stimuli are crucial for efficient inflammatory-immune response because intense and prolonged stimuli cause NETosis, and the resulting NETs could interfere with the recruitment and function of other inflammatory or immunocompetent cells [10].

NET clearance occurs through several mechanisms, mainly through the activity of nucleases [28], because netting PMNs do not release signals characteristic of apoptotic cells, which prevents their elimination by macrophages [36]. Chromatin in NETs is initially cleaved by DNase I, followed by opsonization dependent on a complement C1q fraction, and phagocytosis by macrophages and dendritic cells [65]. DNase I is the main enzyme participating in the NETs breakdown [66]; chromatin can also be degraded by DNase III after endocytosis. NET phagocytosis by macrophages is facilitated through LL-37, a protein with antibacterial activity, which protects NETs from nucleolytic enzymes derived from bacteria [65,67]. Recently, T-series resolvins (RvTs) with important roles in resolution of inflammation have been demonstrated to promote NET clearance by macrophages via the cyclic adenosine monophosphate (cAMP)-protein kinase A-AMPK pathway [68].

## 3. Role of NETs in Periodontitis Initiation and Progression

PMNs play key roles as both effectors of the innate immunity against periodontal pathogens and as host factors responsible for promoting collateral tissue damage. Under normal conditions, PMNs are found in the oral mucosa, saliva, and gingival crevicular fluid (GCF) to survey the periodontal tissues and to ensure local immunological homeostasis.

The morphology of gingival sulcus, particularly the narrowness, which maintains close contact with the sulcular epithelium and the subgingival biofilm, facilitates the onset and progression of gingivitis and periodontitis. On the other hand, the permeability of junctional epithelium allows for the continuous routing of PMNs from the gingival blood vessels into the sulcus to provide protection against periodontal pathogens in the dental plaque. Schiött and Löe demonstrated that salivary leukocytes originate from the gingival sulcus, at a rate of about 30,000 leukocytes/minute. The authors also reported that the number of salivary leukocytes varied between individuals and during the day. Additionally, it depended on the periodontal health status: under normal conditions, the number of leukocytes was small, but during development of gingivitis, the number increased progressively, from 5 to 17 times [69]. PMNs’ migration through the oral epithelium at sites of microbial infections, particularly into the gingival sulcus to control the subgingival dental biofilm, is mediated by the expression of adhesion molecules and local release of chemotactic cytokines. Tonetti et al. demonstrated the expression of intercellular adhesion molecule-1 (ICAM-1) receptors and IL-8 in the epithelial cells making up the junctional epithelium, with high levels particularly in the superficial cells that come into contact with the bacterial biofilm. These molecular mechanisms ensure PMNs’ efficient transepithelial migration into the gingival sulcus, playing crucial roles in maintaining the local host–pathogen balance [70]. Quantitative deficiencies and qualitative defects in PMNs due to alterations in the innate or acquired immune response increase predisposition to aggressive forms of periodontitis. Maintaining periodontal homeostasis is therefore crucial for oral health.

Upon activation by various stimuli, PMNs migrate by chemotaxis at the site of bacterial invasion and activate the complex mechanisms of their defense arsenal, including NET formation [71,72]. A particular aspect regarding PD, which is different from other pathologies implicating NETosis, is that the interaction between PMNs and pathogenic bacteria occurs mainly outside the tissues, in the gingival sulcus [73,74]. Moreover, PMNs in the oral tissues exhibit particular chemotactic and antibacterial activity compared to circulating PMNs [75]. PMNs interact with biofilm bacteria by various intricated mechanisms. Primarily, PMNs aim to destroy pathogens, but certain bacteria can evade PMNs’ defense arsenal [6].

Various dysfunctions of PMNs have been associated with PD. Thus, PMNs’ defective recruitment and extravasation [76] have been reported to reduce chemotactic activity and to prolong transit time [77], which are pathogenic mechanisms leading to persistent inflammation and PMNs-associated tissue damage in PD [44,78]. In older people, peripheral PMNs are less responsive and NET formation is reduced; this observation could explain the increased PD prevalence associated with aging [76], since individuals with decreased levels of NET are susceptible to tissue invasion by periodontopathogenic bacteria, and are at risk of developing periodontitis [10].

The periodontal infectious–inflammatory reaction involves a complex interplay of pro-inflammatory cytokines and adhesion molecules, resulting in the massive recruitment of PMNs in the periodontal tissues, and the dysregulated activation of PMNs in response to periodontal pathogens in the subgingival biofilm [10]. Previous investigations have shown that PD leads to hyperactivity and hyper-reactivity of peripheral blood PMNs, possibly due to abnormal cell phenotype or activation by increased levels of proinflammatory cytokines [79,80]. Hyperactive PMNs produce ROS without the presence of exogenous stimuli, whereas hyper-reactive PMNs generate excessive amounts of ROS upon stimulation [81]. Thus, the exaggerated responsiveness of periodontal PMNs results in increased adhesion, generation of ROS, proteolytic enzymes, and NETs, leading to enhancement of inflammation with detrimental effects due to collateral tissue damage [44,81,82].

In patients with chronic PD, high levels of specific enzymes, including MPO and matrix metalloproteinases (MMPs), of which the most relevant are MMP-2, MMP-8, MMP-9, and MMP-13, were found in the peripheral blood, gingival epithelium, and GCF associated with persistent inflammation and tissue degradation [72,83,84,85,86].

Of relevance to PD, the presence of NETs was reported in the subgingival biofilm [87], in the gingival crevice and associated with the sulcular epithelium [88], and deeper in the periodontal tissues [57]. Increased amounts of NETs can be interpreted as a local defense mechanism against periodontal pathogens and a compensation to overwhelmed phagocytosis [73,88,89]. However, NETs’ effectiveness could be insufficient in the case of massive bacterial invasion [6]. NET formation can be stimulated by both gram-positive and gram-negative bacteria in the biofilm, such as: *Porphyromonas gingivalis* (*P. gingivalis*), *Aggregatibacter actinomycetemcomitans* (*A. actinomycetemcomitans*), *Fusobacterium nucleatum*, *Prevotella intermedia* (*P. intermedia*), and *Veillonella parvula* [10,14]. Hirschfeld et al. demonstrated that PMNs are attracted into dental plaque and are stimulated by the periodontal pathogenic bacteria to release NETs and AMPs, thus, PMNs are responsible for controlling the microorganisms in the biofilm. Furthermore, the variability in NET formation observed among patients could explain the different evolution of PD [90]. On the other hand, periodontal pathogens possess multiple mechanisms, enabling them to elude NETs, and thus promoting tissue colonization, exacerbated inflammatory response, and onset of periodontal breakdown.

**Figure 2:**
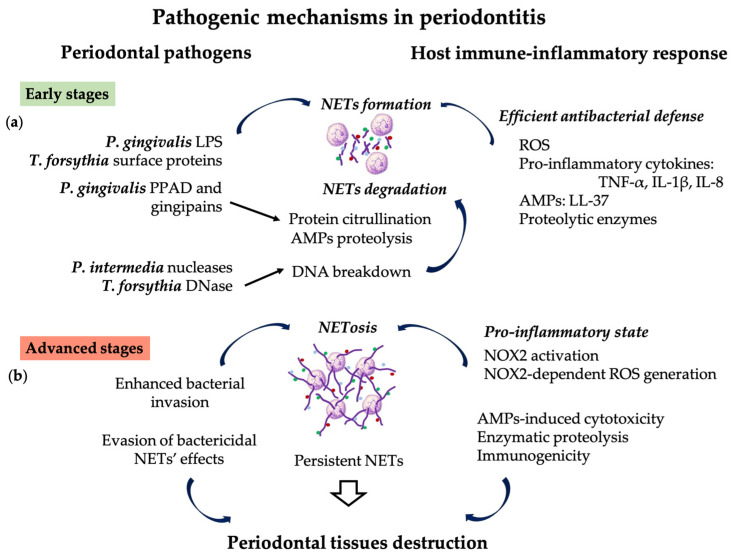
Pathogenic mechanisms involving NET formation and NETosis in periodontitis. (**a**) Early stages of PD: periodontal pathogens express various virulence factors such as LPS and surface cell proteins that induce NET formation, and factors that degrade NETs, such as PPAD, gingipains, and nucleases, allowing bacteria to evade the NETs; host-derived factors in the periodontal tissues, including ROS, IL, TNF-α, and AMPs enable efficient antibacterial defense. (**b**) Advanced stages of PD: enhanced bacterial invasion and NOX2-independent ROS formation induce NETosis and maintain pro-inflammatory state; persistent NETs and immunogenicity lead to periodontal destructions.

Dental biofilm is a complex organization of microorganisms residing in an extracellular matrix, comprising both pathogenic and non-pathogenic species which interact with each other in order to maintain a balance between the plaque microbiota and the host’s immune system [91]. Socransky et al. classified biofilm bacteria according to their pathogenicity, and defined five specific color codes assigned to bacterial communities: (i) the red complex including pathogenic bacteria present mainly in periodontitis patients: *Porphyromonas gingivalis* (*P. gingivalis*), *Tannerella forsythia* (*T. forsythia*), and *Treponema denticola*; (ii) the orange complex including species often found in periodontitis: *Fusobacterium*, *Prevotella* and *Campylobacter*; (iii) the yellow complex containing the *Streptococcus* species; (iv) the green complex containing the *Capnocytophaga* species; and (v) the purple complex containing species such as *Veillonella parvula*, *Actinomyces odontolyticus*, and *Actinomyces naeslundii*, usually found in healthy individuals [92]. Numerous species, particularly pathogens in the red, orange, and yellow complexes of the dental biofilm, release extracellular DNase and different nucleases, enzymes capable of degrading DNA in the structure of NETs [6,93] (Figure 2a).

*P*. *gingivalis*, one of the main periodontal pathogens, expresses virulence factors such as gingipains and peptidyl arginine deiminase (PPAD), which are responsible for local lytic activity in the periodontal tissues, alteration of the immune response, and exacerbation of inflammation [94,95]. Gingipains are cysteine proteinases expressed on the *P. gingivalis* outer membrane that promote bacterium adherence to the host cells, tissue invasion, subsequent lysis of the extracellular matrix, and resorption of the alveolar bone [96,97,98]. PPAD is an enzyme unique to *P. gingivalis* among prokaryotes, capable of converting arginine into peptidyl citrulline, and altering the function of host proteins such as AMPs and histones [95]. By protein citrullination in the periodontal tissues, gingipain R (Rgp), in conjunction with PPAD, reduces the efficiency of NETs and enables bacterium to evade the host immune defense [4,94]. Furthermore, by protein citrullination in other remote sites, including in the synovial membrane, atherosclerotic plaques and nerve cells Rgp and PPAD are implicated in the pathogenesis of systemic diseases, such as: RA, diabetes mellitus, atherosclerosis, SLE, and neurodegenerative diseases [94,95]. LPS, the endotoxin associated with the cell wall, can initiate NET formation either directly or indirectly via circulating platelets. TLR4 expressed by platelets enable the recognition of LPS derived from gram-negative periodontal pathogens [99,100]. Still, due to the proteolytic activity of gingipains, the AMPs in the NETs are degraded, and the antibacterial function of the NETs is altered [97].

*P. intermedia* exerts high DNA-degrading activity, enabling bacteria to escape being killed by NETs [101], hence the paradox related to microbial pathogenicity: even though their virulence factors induce PMN recruitment and NET formation in the periodontal tissues, periodontal pathogens can evade the bactericidal effect of NETs and thrive in the periodontal pockets [97].

*T. forsythia* is associated with severe forms of periodontitis, and uses survival adaptation and activates various factors to induce pathogenicity to evade the host’s defense. Virulence mechanisms involve sialidase and proteases to degrade proteins, outer membrane glycoproteins which enable bacterial invasion, and adherence and surface LPS that trigger inflammation. Furthermore, through its interaction with oral epithelium and inflammatory cells in the periodontium, *T. forsythia* is able to colonise periodontal tissues, to alter the immune response, and to promote tissue destruction [102]. Cell-specific responses induced by *T. forsythia* include: increased tissue invasion by attaching to oral epithelial cells and dental follicle mesenchymal stem cells, ROS formation upon phagocytosis of non-opsonized *T. forsythia* by PMNs, and the alteration of PMN functions: reduced chemotaxis and phagocytosis, and NET formation. As a common trait in other periodontal pathogens in the red complex of the dental biofilm, *T. forsythia* was reported to produce DNase to destroy the NETs and to evade the PMNs’ defensive mechanism. Such dysregulations in the PMNs’ activity may promote the persistence of oral pathogens [102].

*A. actinomycetemcomitans*, a gram-negative bacterium common in patients with aggressive periodontitis, is capable of attaching to the gingival epithelium, further invading periodontal tissues and producing leukotoxin which destroys the host’s inflammatory cells [91].

Moreover, the particular conditions within periodontal pockets promote increased ROS generation and NOX-dependent NET formation that could exert deleterious effects [10,73]. Proinflammatory cytokines involved in the host’s response against periodontopathogens, including TNF-α, IL-1β, and IL-8, can induce NET formation [103]. In particular, LL-37 AMP present in the GCF, in addition to subgingival biofilm, could represent a stimulus for PMN activation and NET formation. LL-37 is a 37-residue peptide, a member of the linear or α-helical AMPs family, and the only cathelicidin described in humans which performs antimicrobial activity against a broad spectrum of bacteria. The name of this AMP derives from its structure consisting of 37 amino acids, of which the first two are N-terminal leucines (LL) [104]. LL-37 is secreted mainly by PMNs, but also by other cells in the periodontal tissues, such as monocytes and epithelial cells, and can be detected in GCF in both healthy individuals and periodontitis patients [105].

In the early stages of periodontal disease, NETs exhibit more intense bactericidal activity due to citrullinated histones, mainly citrullinated histone 3 (H3Cit) [106]. Then, as gingivitis progresses to PD, H3Cit plasma levels decrease because histones are degraded by MPO and other PMN enzymes [38,106]. Several studies have reported that the presence of NETs activates new PMNs recruited at the inflammatory site for an efficient antibacterial defense [22,24,64].

However, as the lesion matures, PMNs die via NETosis [44] and the accumulation of NETs in the periodontal tissues maintains a pro-inflammatory state [10,22,29,107] and initiates amplifying pathways by further NET formation [22,24,63,64]. These pathways involve recognition of NETs by TLR8 and TLR9 receptors on the PMNs, followed by NOX2 activation, NOX2-dependent ROS generation, cytotoxic effects exerted by AMPs, and immunogenicity by the presence of citrullinated histones and extruded DNA strands [10,22,29,63,64,107]. Additionally, the interconnection between NETs and acquired immunity by the priming of T cytotoxic lymphocytes to release proinflammatory mediators could be another pathway contributing to periodontal breakdown [108] (Figure 2b).

Even though NETs are essential defense mechanisms against periodontal pathogens, their timely clearance is crucial for preventing host tissue damage mediated by the AMPs, auto-immunogenic DNA, and remaining bacterial debris [10]. NETs clearance is initiated by the degradation of DNA by host- and bacteria-derived DNases [109]. It has been reported that, in chronic diseases, the sustained immune stimulation results in the production of anti-NETs immunoglobulins and particularly excessive free light chains (FLCs) that form a physical barrier to protect NETs from enzymatic degradation [110]. FLCs are produced by B lymphocytes, the predominant type of immunocompetent cells in the active PD sites [8]. In PD patients, the presence of serum autoantibodies against citrullinated and non-citrullinated histones was reported [111]. Similarly, White et al. demonstrated significantly lower DNase I plasma levels and increased circulating levels of IgG and FLCs, consistent with reduced NET degradation activity, in PD patients before treatment. However, NET production decreased and NET degradation improved after successful periodontal treatment [8]. Increased NET accumulation by both excessive formation and impaired elimination is a possible pathogenic mechanism leading to PMNs-mediated periodontal tissue damage [77].

In susceptible PD patients, the interaction between dysfunctional PMNs and pathogenic bacteria results in an exaggerated immune response and prolonged inflammatory state, ultimately causing tissue injury [81]. Thus, increased NETosis and excessive formation of NETs in response to constant invasion by pathogens of periodontal tissue, the ability of microorganisms to evade NETs, and the inefficient removal of NETs are mechanisms promoting disease progression and severity [10]. Therefore, NETosis could be a novel paradigm in PD pathogenesis.

## 4. NETosis Biomarkers, and Diagnostic Tools in Periodontitis

Currently, there is a paucity of studies on molecular diagnosis, the development of reliable diagnostic tools, and the efficient treatment in PD. A better understanding of the role of NETs, the molecules associated with the immune-inflammatory host response, and the pathogenic pathways in PD could provide markers useful for the diagnosis and monitoring of disease progression, and for assessing treatment efficacy. Since increased NETosis can lead to the onset and progression of various diseases, including PD, NET biomarkers could be considered useful tools in the diagnosis and monitoring of these diseases [112].

### 4.1. Biomarkers Related to NET Formation

Increased NET formation is associated with the hyperactivity of NOX, thus, the expression of isoforms and subunits of enzymes, such as NOX1, neutrophil cytosolic factor 2 (NCF2), Rac1, and Rac2, could be assessed for monitoring NETosis during PD progression. NCF2, an intracellular protein representing a subunit of the NOX enzyme complex, has low levels under physiological conditions. Increased levels of NOX1 and NCF2 could indicate higher oxidative activity associated with NETosis [113]. Rac1 and Rac2 are NOX complex subunits also implicated in cytoskeleton formation, maintaining cell shape and regulating PMN functions such as adhesion and migration. Histone citrullination is dependent on Rac1, Rac2, and the downstream effector molecule serine/threonine-protein kinase Pak2 [114]. Thus, H3Cit, which is essential for NET formation, could be considered an early marker of subsequent NETosis [115].

### 4.2. Biomarkers Related to NET Accumulation

These biomarkers could indicate the severity of PD in the context of NETosis-related tissue destructions and the therapeutic success of arresting periodontal breakdown caused by NETosis. Diagnostic tests using only cell-free DNA (cfDNA) to assess NETosis are non-specific, and DNA should be considered together with other NET components, such as histones and/or NE, since NE indicates that DNA derives from the de-condensation of nuclear chromatin of PMNs [56]. NETs’ efficient degradation within a short span of time is essential for preventing tissue damage [113]. Therefore, DNase I, the enzyme implicated in NET degradation, could be investigated for assessing the efficient removal of NETs during periodontal treatment and regression of local inflammation.

### 4.3. Associated Non-Specific Biomarkers

The inter-relation between inflammation, oxidative stress, and NETosis provides biomarkers of inflammatory disease states, such as: NF-kB, pro-inflammatory cytokines, NO, plus oxidation-related products, such as MDA, protein carbonyls, AGEs, 3-nitrotyrosine, and 8-oxoguanine, which are also useful in PD diagnosis.

Previously, higher salivary MPO concentrations were reported in patients with chronic PD compared with healthy controls and gingivitis patients [116,117]. Even though MPO is a component of NETs and implicated in NET formation, it is not a reliable marker for NETosis. Firstly, NET formation can occur in the absence of MPO, in response to bacteria-derived stimuli [38,50]. Instead, NE is the predominant enzyme that induces chromatin de-condensation and NET formation in vivo [47]. Secondly, increased MPO levels can be the result of regular, NET-independent PMN degranulation. Therefore, for reliable diagnostic value, only MPO attached to DNA should be assessed, as a marker for NETosis [29].

### 4.4. Systemic versus Local Biomarkers

Circulating NET biomarkers such as PAD4, extracellular DNA, MPO, NE, and nucleosomes can be assessed in plasma and serum, but the disease- and origin-related specificity could be uncertain, especially in patients with multiple co-morbidities [118]. Therefore, levels of biomarkers assessed in saliva or GCF could be more specific in reflecting the local formation of NETs, and more valuable for PD diagnosis and monitoring. Increasing evidence suggests saliva is an alternative to classical biofluid assessment due to multiple advantages and reliability as a diagnostic tool [119]. The quantification of salivary biomarkers including cell-free nucleosomes, MPO, and NE are reliable tools for assessing NET formation in the oral tissues [12,120].

### 4.5. Novel Tools for Assessing Biomarkers in Oral Biofluids

The gold standard technique used for the detection of MPO, NE, and nucleosomes is the traditional enzyme-linked immunosorbent assay (ELISA) [113,121] Even though this method has commercially available kits with high sensitivity and specificity, several disadvantages have been described that new methods of analysis, especially the ones based on electrochemical sensors, are trying to overcome. Novel electrochemical sensors are suitable for the development of integrated, portable, and wearable sensing devices, which can easily facilitate the determination of biomarkers, including these cytokines in biological fluids, with applicability in medical practice [122,123]. The use of aptamers that specifically bind the complementary analytes enables the development of aptasensors for the specific detection and quantification of the selected targets [124,125], which could enable the detection of NETosis biomarkers in saliva.

Numerous experimental and clinical studies reported a correlation between bodily fluid levels of NET biomarkers, such as: H3Cit, MPO, NE, cfDNA, nucleosomes, and the severity of various diseases, including thrombosis, autoimmune diseases, infectious diseases, and periodontitis [72,118].

The intricate relations between NETosis, chronic periodontal inflammation, and tissue destruction suggests the need for using multiple biomarkers to assess disease severity. Investigating the correlation between levels of different components of NETs, specific enzymes, and cytokines in oral tissues and biofluids could enable the validation of new biomarkers in PD [72]. Accurate diagnosis and prognosis should be based on the assessment of complex panels of biomarkers that could comprise both markers for NETosis and biomarkers already validated by experimental and clinical studies, such as MMP-8. Biomarkers capable of predicting PD onset and progression could ideally serve for early diagnosis, timely and efficient intervention, and personalized therapy [72].

## 5. NETs as Potential Therapeutic Targets in Periodontitis

Although the inflammatory response against periodontal pathogens is protective, the failure to remove noxious products resulting from PMNs’ phagocytotic activity, delayed apoptosis, and the incomplete clearance of apoptotic inflammatory cells leads to pathological changes characteristic of chronic periodontal lesions. Growing data suggest that PD may be a risk factor for various systemic conditions, such as cardiovascular diseases, diabetes, Alzheimer’s disease, RA, adverse pregnancy outcomes, and cancer. Therefore, there is an urgent need to develop effective therapies and preventive approaches by targeting the main pathogenetic mechanisms [13]. Given the determining role of the infectious factor in periodontitis pathogenesis, conventional periodontal therapy aims to reduce the microbial load. The non-surgical periodontal treatment consists of complex procedures involving debridement, scaling to remove subgingival calculus, and dental plaque, and root planing to remove endotoxin-impregnated cementum from the root’s surface. In addition to the manual instrumentation of periodontal pockets, the local application of various antiseptics, antibiotics, and anti-inflammatory agents is recommended [126].

**Figure 3:**
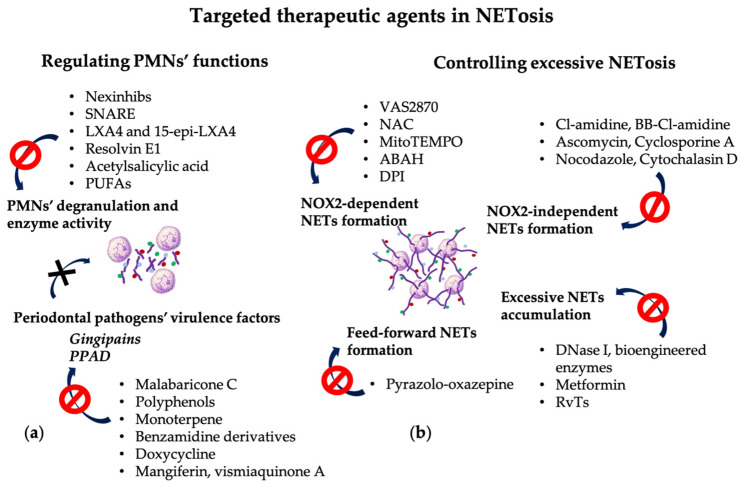
Emerging targeted therapeutic agents with applicability as LDD systems in NETosis. Strategies aim to regulate physiological PMN functions and to control the deleterious effects of excessive NETosis. (**a**) PMN functions can be restored through several pathways: preventing enzyme secretion, suppressing degranulation and controlling PMNs’ tissue infiltration, reducing the formation of ROS, and the release of pro-inflammatory cytokines. Detrimental effects of periodontal pathogens can be prevented by using phytochemicals or chemical compounds to inhibit the gene expression and activity of gingipains and PPAD. (**b**) Excessive NET formation can be controlled by different means, depending on the pathogenic mechanism of NETosis: NOX2-dependent NET formation can be counteracted by using NOX2 inhibitors or antioxidants, whereas NOX2-independent NET formation can be controlled by inhibiting PAD4, histone citrullination, the calcineurin pathway, and the cytoskeleton rearrangement. Excessive NET accumulation can be prevented by increasing extracellular DNA degradation, PMNs’ efferocytosis, and NET phagocytosis. Feed-forward NET formation can be prevented by controlling GSDMD cleavage, pyroptosis, and inflammasome activation.

Since PMNs play a central role in periodontal inflammation, novel therapeutic strategies should focus on controlling PMN functions to prevent NETs-mediated collateral damage and/or promoting the timely clearance of PMNs and NETs from the periodontal tissues after the fulfillment of their function [15] (Figure 3).

Numerous studies have investigated the efficacy of therapeutic approaches aimed at blocking various molecules associated with NET formation. However, not all the molecules implicated in the NET pathway can be therapeutic targets because the inhibition of key factors could be detrimental for the physiological functions of PMNs [56].

### 5.1. Targeting PMNs’ Degranulation and Enzyme Activity

Experimental animal models of various pathologies have demonstrated the efficacy of pharmacological agents used to control PMNs’ degranulation [127,128,129] and to reduce enzyme activity [130,131,132] (Table 1).

Molecular inhibitors that alter PMNs’ secretory activity include: neutrophil-specific exocytosis inhibitors (Nexinhibs), which prevent the exocytosis of azurophilic granules enzymes [127,128] and soluble N-ethylmaleimide-sensitive factor attachment protein receptor (SNARE) mimetic aptamers with selectivity for PMN granules, which can control vesicular transport and granule secretion [127].

MPO catalyzes the generation of hypochlorous acid, an oxidant with an increased cytotoxic effect, and also signals through β2-integrin Mac-1 to prevent PMN apoptosis. Lipoxin A4 (LXA4) and 15-epi-LXA4 are anti-inflammatory lipids formed in PMNs and other cells under the influence of acetylsalicylic acid (Aspirin) and statins (Atorvastatin), which suppress MPO-induced Mac-1 expression and promote the resolution of inflammation [132]. Animal models for PD demonstrated that increased levels of endogenous LXA4 and resolvin E1 had a protecting role by controlling PMN tissue infiltration and preventing bone loss [133,134]. Used for in vitro studies on human PMNs, LXA4 and resolvin E1 reduced the formation of ROS in response to TNF-α and bacterial-derived peptides [135]. Dietary supplementation with acetylsalicylic acid and ω-3 polyunsaturated fatty acids (PUFAs) in PD patients previously treated by conventional scaling and root planing improved clinical parameters and decreased salivary levels of pro-inflammatory cytokines [136].

### 5.2. Targeting the NOX2-Dependent NET Formation

Since NET formation occurs mainly through the NOX2-dependent pathway, a promising approach could be to reduce NETosis by targeting NOX2 and by controlling ROS generation and scavenging [28,36]. Dömer et al. demonstrated that VAS2870 inhibited NOX2 activity and the subsequent NET formation [22]. However, some problems related to this approach have been reported because the disruption of NOX and oxidants could lead to an increased risk of infection and autoimmunity [56]. Animal models in which the NOX complex was inhibited resulted in more severe RA and SLE [148], and patients with a genetic defect of NOX2 had an increased risk of autoimmune conditions [149]. Antioxidants such as N-acetyl cysteine (NAC) proved to be efficient for SLE evolution [137], and specific scavengers, such as MitoTEMPO, used to neutralize mitochondrial ROS, reduced pro-inflammatory cytokines, mainly type I interferon, and improved disease progression in a lupus animal model [138]. An in vitro study demonstrated that inhibitors of NOX, e.g., diphenyleneiodonium (DPI), and inhibitors of MPO, e.g., 4-aminobenzoic acid hydrazide (ABAH), blocked the formation of free radicals mediated by NO donors [58].

### 5.3. Targeting the NOX2-Independent NET Formation

Another potential target for inhibiting NETosis without affecting oxidative activity is PAD4. PAD4 inhibitors prevent histones’ citrullination, disrupting the cascade of DNA de-condensation, and decreasing the intensity of “suicidal” NETosis [150,151,152]. Pan-PAD inhibitors, such as N-α-benzoyl-N5-(2-chloro-1-iminoethyl)-L-ornithine amide (Cl-amidine) or BB-Cl-amidine, were efficient in animal models for autoimmune diseases, but an increased risk of infections was reported as a side effect [56,139].

Studies have reported that extra- and intracellular calcium pools are essentially implicated in NET formation in various pathologies, suggesting that antagonists of the calcineurin pathway, such as Ascomycin and Cyclosporine A, can be used to control NETosis [39].

The reorganization of cytoskeletal components essentially enables chromatin extrusion, thus, inhibitors of tubulin polymerization and actin filamentation, such as Nocodazole and Cytochalasin D, have proven effective for preventing NET formation in response to LPS [37].

### 5.4. Targeting Excessive NET Accumulation

Excessive NETs, as a consequence of increased NETosis or inefficient clearance, may contribute to tissue damage. Therefore, innovative therapeutic approaches in PD aim to develop molecular agents capable of controlling NET formation, without interfering with PMNs’ essential function in controlling bacterial invasion. Moreover, strategies for preventing excessive “lytic” NETosis, limiting NETosis-associated tissue damage, and promoting the resolution of periodontal inflammation could allow for the better monitoring of PD. Two approaches could be used for improving NET clearance: DNase-induced degradation and elimination by macrophages. The most convenient therapeutic agent is, probably, the DNase. This enzyme does not inhibit NET formation, but is capable of disrupting NETs’ structure by hydrolysis of the extracellular DNA. Moreover, DNase could delay the formation of autoantibodies against extracellular DNA, thus reducing the autoimmunity-associated pathologies. Recent therapies aiming to increase NET degradation are based on recombinant human DNase I and other bioengineered enzymes that have been proven to degrade extracellular DNA, the main component of NETs and DAMPs [140]. The administration of DNase I in animal models was beneficial, but in patients with SLE, recombinant DNase did not induce a significant effect on serum markers or disease evolution. These results could be due to fact that autoantibodies were already present at the time of the treatment application and had been formed prior to NETs’ removal [153]. The advantages of DNase administration reside in the effective NETs’ lysis, therapeutically efficient levels in serum after intravenous administration, minimal adverse effects, and the absence of neutralizing antibodies against DNase [153,154]. Important limitations of DNase-based therapy are reduced stability and rapid clearance. Therefore, Wang et al. proposed nanozymes, obtained by the immobilization of DNase on the surface of polymeric nanoparticles to ensure optimal delivery and prolonged therapeutic activity in inflammatory bowel disease [154]. Another approach is to ensure efficient PMN efferocytosis in order to limit the exposure of surrounding tissues to NET components and to prevent extensive collateral damage. The activation of AMP-activated protein kinase (AMPK) plays essential roles in enhancing macrophages’ phagocytic activity, resulting in a potent anti-inflammatory effect [155]. Grégoire et al. reported that Metformin, a biguanide anti-hyperglycemic agent, could also be used as an activator of AMPK to improve PMNs’ efferocytosis and NET clearance in patients with acute respiratory distress syndrome (ARDS) [48]. Due to their function in NET phagocytosis by macrophages, RvTs, particularly RvT2, could be considered as a possible strategy to monitor NET clearance and to control NET-induced collateral tissue damage [68].

### 5.5. Targeting the Feed-Forward Mechanism of NET Formation

Therapeutic approaches should also counteract the implication of NETs in the further auto-amplification mechanism of inflammation. Molecules such as Pyrazolo-oxazepine are capable of controlling GSDMD cleavage, pyroptosis, and downstream inflammasome activation [60]. Several studies have reported that, at the site of the inflammation, exposure of newly recruited PMNs to previously formed NETs amplified their pro-inflammatory activity, resulting in the generation of ROS, the formation of ROS-dependent NETs, and stimulated phagocytosis [22,26,64]. Therefore, therapeutic approaches addressed at NET clearance should also prevent secondary NETosis. It is still unclear what signaling pathway is activated by the interaction between TLR8 and TLR9 receptors and NET components that leads to secondary NETosis; further investigations could indicate the key component of NETs and the engaged receptor capable of triggering the cascade of NETs-induced NETosis [22].

### 5.6. Targeting Periodontal Pathogens’ Virulence Factors

The pharmacological targeting of virulence factors derived from periodontal pathogens, putatively for NETosis, could also be an effective approach. Natural or synthetic compounds capable of reducing the proteolytic activity of gingipains and the citrullination potential of PPAD could be a promising strategy for managing both PD and systemic conditions [94]. Plant-derived compounds controlling the gene expression of gingipains or with an inhibitory effect on the proteolytic activity of gingipains include: malabaricone C derived from nutmeg [141], polyphenols—catechin derivates [143], and monoterpene from *Nigella sativa* (black cumin) [143]. Chemical compounds effective as gingipain inhibitors include: benzamidine derivatives [144] and common antibiotics, such as tetracyclines, particularly Doxycycline [145,146]. Tan et al. reported that mangiferin and vismiaquinone A contained in *Cratoxylum cochinchinense* extract could be inhibitors of PPAD [147].

### 5.7. Targeting Other Related Pathogenic Mechanisms

In order to counteract the oxidative and nitrosative stress associated with periodontal chronic inflammation, the human body has several endogenous antioxidant mechanisms, both enzymatic (e.g., superoxide dismutase, catalase, and glutathione peroxidase) and non-enzymatic (e.g., lipoic acid, glutathione, ʟ-arginine, and coenzyme Q10—Co-Q10). Beside these, multiple antioxidants of exogenous origin, either animal or plant-derived molecules, can be added to the diet as nutritional supplementation [156] or can be administered by local applications [157].

The intricate mechanisms involving NETosis in PD pathogenesis highlight the importance of developing novel therapies aimed at controlling the excessive PMN recruitment and activation in the periodontal tissues [6].

## 6. Local Drug Delivery Systems as Novel Periodontal Therapeutic Approaches

In periodontal therapy, LDD systems are designed to be more targeted for enhanced efficacy, minimal side effects, and optimized bioavailability to surrounding tissues. Moreover, therapeutic agents can be delivered into the periodontal pocket in a predictable and controlled manner, and maintained for an adequate amount of time for the therapeutic effect to occur [158].

Methods using mouthwashes, gels, toothpastes, and supra- and subgingival irrigations fail to maintain therapeutic concentration in the periodontal pocket and require higher initial concentrations and/or multiple applications to provide sustained effectiveness. To circumvent these drawbacks, controlled and prolonged release LDD systems have been developed to enhance pharmacokinetics, improve drug loading and stability, and provide specific targeting [126,159].

### 6.1. Required Properties of LDD Systems

The required features for an intra-pocket LDD system include: mucoadhesivity, biocompatibility, non-toxicity, biodegradability, and protecting the active therapeutic molecule from premature degradation [126].

The mechanical properties of LDD systems designed for application in the periodontal pocket have a pivotal role in clinical performance. The increased consistency of the system can ensure an adequate mechanical resistance to the structural mixture of the formulation and influence the release kinetics of the APIs through erosion or diffusion from it. Ensuring an adequate viscosity allows, on the one hand, an optimal flow that enables the easy and non-invasive application of semi-solid products, but also promotes the formation of the bioadhesive bond [160,161]. Obtaining resistant but flexible films after swelling with saliva is an important goal in formulation, as it allows the attachment of the product to the gingival mucosa and ensures the sealing of the periodontal pocket. It is also necessary that the vehicles used in the formulation of periodontal LDD systems allow the incorporation of high concentrations of active ingredients, considering the small applicable volume in the periodontal pocket [159].

*Mucoadhesivity* is an essential property of the LDD systems that ensures prolonged maintenance at the site of application and the intimate contact of therapeutic agents with the oral tissues for improved absorption and bioavailability. In order to promote adhesion of the macromolecules to the oral epithelium, the characteristic properties of the oral mucosa should be taken into consideration: the local temperature, the secretion of saliva and the outflow of inflammatory exudate or GCF in the periodontal pocket, the composition of these biofluids, and the mechanical functional forces that could dislodge the LDD system. The mucoadhesion on a larger surface area promotes the close contact for better diffusion into the tissues and increased attraction forces to the substrate [162,163]. A partly hydrated polymer is attracted and connects better with a humid surface through the activation of mucoadhesive compounds in the presence of the water. Moreover, the plasticity of the formulation occurs due to moisture and promotes van der Waals interactions between molecules, resulting in covering and swelling [164].

*Biocompatibility, non-toxicity, and biodegradability* are properties related to the behavior of a biomaterial in different contexts and refer to the ability to decompose in a controlled manner through the interaction with living tissues, without generating toxic byproducts that could exert deleterious effects [165,166]. The degradation rate and controlled release dynamics are essential factors influencing the delivery of bioactive agents to the targeted tissues and the therapeutic outcome. Sequential administration of multiple active pharmaceutical ingredients (APIs) with additive or synergistic effects can be achieved by controlling the time frame for release of the adsorbed and/or incorporated molecules, depending on the intended effect [167,168].

The development of different formulations of LDD should take into consideration various factors, including: (i) the degradation mechanism: hydrolytic or enzymatic; (ii) the persistence time necessary for releasing the APIs; (iii) the physico-chemical properties, such as: porosity, hydrophilic, or hydrophobic nature, molecular weight, and density [166,169].

### 6.2. LDD System Constituents and Formulations

Biomaterial constituents and formulations must be chosen according to the composition of the APIs to stabilize the bioactive molecules and to prevent unwanted interactions that could lead to alterations of the therapeutic potential. Currently, controlled release devices with great potential for various biomedical applications are available in the form of hydrogels, emulgels, films, tablets, nanofibers, and patches [158].

*Hydrogels* are polymeric compounds with a three-dimensional conformation, capable of retaining a large amount of water or biofluids, but do not dissolve. Due to their hydrophilic nature, hydrogels do not interact with proteins and cells at the application site [170,171]. Hydrogels can be obtained from various synthetic and natural polymers, and their properties derive from the molecular subunits in the polymeric chain. By chemical modifications of the molecular units, the functional properties of the hydrogels, such as rigidity, swelling ratio, and processing, can be adjusted according to the clinical purpose. Due to their inherent properties, or through chemical changes, hydrogels can respond to thermal stimuli and pH at the site of delivery by swelling or by biodegrading [170].

*Stimuli-responsive hydrogels* represent a promising platform for the topical release of drugs in the periodontal pocket, promoting periodontal regeneration [172,173]. These hydrogels are based on SMART polymers, capable of forming fluid colloidal dispersions at ambiental temperature, but which form gels in situ after coming into contact with the oral tissue, the therapeutic effect being validated both after injection [174] and after topical application [175]. This type of LDD system allows easy application and in a non-invasive manner, being modeled on the shape of the defect, and the formed gel, with a particular geometry, represents a drug reservoir which can ensure the release of active principles with certain kinetics during the period of residence at the level of the periodontal pocket. The gel formation process is time-dependent and varies with the composition of the formulation, mixtures with bioadhesive polymers being preferred, to increase residence at the application site by reducing the erosion rate of the in situ formed gel. The factors involved in the in situ formation of gels are represented by temperature [161], pH [176], light [177], or the presence in the periodontal tissues of specific ions or molecules, such as glucose, reactive oxygen species [178], or enzymes such as MMP-8 [179]. The sol–gel transition occurs by controlling the formation of hydrogen bonds or the appearance of other changes in the hydrogel structure (alteration of charge density, fracture, or crosslinking) [161].

Poloxamers (Pluronics^®^) are considered Smart polymers because they change their structure depending on external stimuli [180], they represent a convenient choice for biomedical applications, and are components of many drug delivery systems, including medical devices. They are non-ionic triblock copolymers formed from polar (ethylene oxide poly) and non-polar (propylene oxide poly) units, a fact that gives them amphiphilic and surfactant properties, being able to incorporate a wide range of active molecules (hydrophilic, lipophilic).

They are water-soluble, non-toxic, non-irritating, FDA-approved, and fulfill multiple roles in pharmaceutical systems (solubilizer, mold former, humectant, emulsifier, stabilizer) intended for oral, parenteral, and topical administration. Aqueous dispersions undergo a sol–gel transition upon increasing temperature, a reversible process that leads to the self-aggregation of molecules into micelles, nanostructures between 10 and 200 nm, which encapsulate/release active molecules [161,181]. Gelation of mixture poloxamers—different polymers after contact with oral fluids confirmed the drug reservoir formation that ensured a controlled release of drugs, demonstrated by various studies [160,175,182,183,184].

*Emulgels* are combined formulations of gels and emulsions, and can serve, together with oleogels, as topical release systems of lipophilic molecules. The use of materials that optimize tissue adhesion is required, considering the easier elution due to the lipid component [185,186].

*Mucoadhesive oral biofilms* have additional advantages resulting from physical properties such as flexibility, elasticity, adhesion to the oral mucosa, and good tolerance. Biofilm preparations can be based on synthetic or natural polymers, or mixtures of these, to optimize the swelling rate and release dynamics of the loaded APIs [126,159].

Active API-loaded *nanofibers and nanoparticles* are promising LDD systems for a wide variety of conditions. Nanofibers obtained by electrospinning and loaded with antibiotics and hydroxyapatite have been developed to ensure extended antimicrobial and immunomodulatory effects [126,187,188].

Depending on the particular features of the application site, the association of different LDD formulations could enhance the therapeutic success. Andrei et al. demonstrated on an animal model of PD that electrospun nanofibers based on polylactic acid and loaded with Doxycycline had physical properties suitable for application in the periodontal pockets, whereas the mucoadhesive biofilm based on hydroxypropyl methylcellulose and polyacrylic acid was used to seal the gingival surface for retaining the biomaterial in the subgingival area. Furthermore, through the gradual release of the therapeutic agent, the nanofiber biomaterial was efficient at reducing the local activity of metalloproteinase-8 and regulating the systemic levels of pro-inflammatory cytokines [126].

### 6.3. Therapeutic Agents Used in LDD Systems

Although several synthetic antimicrobial, antioxidant, and immunomodulatory agents are used as LDD systems in PD, current pharmaceutical technology focuses on phytotherapy and natural ingredients with minimal side effects, and that are more cost-efficient and equally therapeutically effective. Recent studies have demonstrated the efficacy of bioadhesive delivery systems loaded with antioxidants or anti-inflammatory agents for periodontal treatment [157,189,190].

Phytochemical ingredients have a complex composition and exert multiple activities, hence the difficult identification of specific APIs in a given herbal extract and the mechanisms underlying the therapeutic effects [191]. Regarding the efficacy in controlling NET formation, multiple compounds have been proven to inhibit different pathways in NET formation. For example, flavonoids were demonstrated to block the Raf1-MEK-1-Erk pathway and ROS generation [192], safflor yellow compounds and terpenoids from *Celastrus orbiculatus* significantly blocked NETosis and the formation of NET–DNA complexes, preventing subsequent inflammation and autoimmune reactions [193,194], and a traditional medicine based on *Artemisia annua* downregulated the expression of PAD4 and limited NET formation [195]. Tao et al. reported that salvianolic acid B and 15,16-dihydrotanshinone I contained in *Salvia miltiorrhiza* extract reduced the activity of MPO and NOX, preventing the early formation of NETs [196].

This evidence supports the effectiveness of LDD systems in the treatment of PD, representing a promising area of research for development of novel therapeutic approaches.

### 6.4. Particular Features of LDD Systems for Periodontal Therapy

The delivery of bioactive molecules associated with biomaterials is limited due to the partial or complete degradation and diffusion that occur during the acute phase after application. Therefore, in order to protect the APIs from premature degradation and to ensure sustained release over a longer period of time, biomaterial design strategies are essential to provide the required properties and optimal therapeutic outcomes.

Depending on the intended application, APIs can be associated with various biomaterials either by adsorption or by embedding, depending on the therapeutic purpose, the required dose, and the sequential release of multiple therapeutic agents. Binding or immobilization of therapeutic agents by physical adsorption on the surface of biomaterials [166,167] enables immediate release that could be useful for counteracting and/or neutralizing the virulent bacterial factors that trigger sustained and continuous NET formation. Embedding or including the bioactive molecules in the biomaterial bulk [166] promotes a gradual, slow, prolonged release, with variable diffusion rate, depending on the dynamics of biomaterial degradation. Consistent with these principles, Barabás et al. demonstrated different in vitro kinetics of antibiotic release depending on the association with the nano-substrate. Thus, Doxycycline adsorbed on polylactic acid-based nanofibers was released rapidly in the simulated bodily fluid, resulting in an immediate high concentration, whereas the encapsulated amoxicillin had a slow and sustained release [187].

Therapeutic approaches targeting NETosis in PD should take into consideration the controlled release of the therapeutic agent, since the intended effect is not to inhibit NET formation, but to prevent excessive accumulation in periodontal tissues, to attenuate PMN infiltration into inflamed tissues, and to prevent NETosis.

The use of innovative delivery systems that ensure the prolonged release of APIs with anti-inflammatory, antioxidant, and immunomodulatory activity after topical application could represent a solution for a non-invasive approach in PD. The validation of various therapeutics by in vitro and in vivo research is crucial prior to being implemented in clinical studies. Based on a clear understanding of the pathophysiological mechanisms involving NETosis in PD, the APIs should be specifically targeted against pathogenic factors, because off-targeting drugs may affect local homeostasis and alter the medication response. The possible interactions between the APIs and the components of the LDD systems should also be taken into consideration to prevent inactivation or alteration of the therapeutic agents, with a significant impact on efficacy and response to treatment [94].

## 7. Concluding Remarks and Future Directions

NETs are an essential defense mechanism by which PMNs retain and destroy pathogens, preventing their local and systemic dissemination and inactivating harmful virulence factors. However, growing evidence suggests that aberrant NETs and NETosis, as a result of excessive PMN activation by various pathogens and host-derived factors, lead to a prolonged inflammatory state, collateral tissue destruction, and autoimmunity. Thus, NETosis is an important mechanism underlying the pathogenesis of various systemic diseases, including PD.

A better understanding of the role of NETs, the associated molecules, and the pathogenic pathways of NET formation in periodontal tissues could provide markers of NETosis useful for diagnosis and the monitoring of PD progression, and for evaluating treatment efficacy. The use of saliva and GCF as biofluids for assessing NET biomarkers has the advantage of both a non-invasive collecting procedure and direct contact with periodontal tissues, the site of the inflammatory–immune response against periodontal pathogens. Moreover, the prevention of excessive NET accumulation in periodontal tissues, by both controlling NET formation and their appropriate removal, could be a key for the further development of more targeted therapies. As future perspectives, locally delivered therapeutic agents aimed at regulating NET formation could be associated with conventional periodontal therapy for a multimodal approach, thus limiting the impact of PD on systemic health and providing promising perspectives for optimal disease management and a patient’s quality of life.

## Figures and Tables

**Table 1:** Therapeutic approaches targeting NETs’ formation in periodontitis.

Pathogenic Pathway	Therapeutic Agent	Demonstrated Effect	Type of Study
PMNs’ degranulation and enzyme activity	Nexinhibs	Prevented enzymes’ exocytosis	In vitro study on human PMNs [128]
	SNARE	Controlled granule transport and secretion	Animal models of acute lung injury [129]
	LXA4, 15-epi-LXA4 formed after administration of acetylsalicylic acid and statins	Suppressed MPO-induced Mac-1 expression and controlled PMNs tissue infiltration	In vitro study on human PMNs [133] Animal models of PD [133,134]
	LXA4 and resolvin E1	Reduced ROS formation	In vitro study on human PMNs [135]
	Acetylsalicylic acid and PUFAs	Reduced salivary levels of pro-inflammatory cytokines	Clinical study on PD patients [136]
NOX2-dependent NET formation	VAS2870	Inhibited NOX2 activity	In vitro study on human PMNs [22]
	NAC	Antioxidant activity	Clinical study on patients with SLE [137]
	MitoTEMPO	Neutralized mitochondrial ROS	Animal model of SLE [138]
	ABAH	Inhibited MPO activity and free radicals’ formation	In vitro study on human PMNs [58]
	DPI	Inhibited NOX activity and free radicals’ formation	
NOX2-independent NET formation	Cl-amidine and BB-Cl-amidine	Inhibited PAD4 and histone citrullination	Animal models for autoimmune diseases [56,139]
	Ascomycin and Cyclosporine A	Inhibited calcineurin pathway	In vitro study on human PMNs [39]
	Nocodazole and Cytochalasin D	Inhibited tubulin polymerization and actin filamentation	In vitro study on human PMNs [37]
Excessive NET accumulation	DNase I and other bioengineered enzymes	Degraded the extracellular DNA	Clinical study on ventilated trauma patients [140]
	Metformin	Activated AMPK and PMNs efferocytosis	Clinical study on patients with ARDS [48]
	RvTs	Increased NET phagocytosis by macrophages	In vitro study on human blood [68]
Feed-forward NET formation	Pyrazolo-oxazepine	Controlled GSDMD cleavage, pyroptosis and inflammasome activation	In vitro study on human PMNs [60]
Periodontal pathogens’ virulence factors	Malabaricone C derived from nutmeg	Controlled gene expression of gingipains or inhibited the proteolytic activity of gingipains	In vitro study on *P. gingivalis* culture [141]
	Polyphenols—catechin derivates		In vitro study on *P. gingivalis* culture [142]
	Monoterpene from *Nigella sativa*		In vitro study on *P. gingivalis* culture [143]
	Benzamidine derivatives		In vitro study on *P. gingivalis* culture [144]
	Doxycycline		In vitro study on *P. gingivalis* culture [145,146]
	Mangiferin and vismiaquinone A from *Cratoxylum cochinchinense* extract	Inhibited PPAD	In vitro study on *P. gingivalis* culture [147]

## Data Availability

Not applicable.

## Abbreviations

*A. actinomycetemcomitans*	*Aggregatibacter actinomycetemcomitans*
ABAH-4	Aminobenzoic acid hydrazide
A-AMPK	Cyclic adenosine monophosphate-protein kinase
AGEs	Advanced glycation end-products
AMPs	Antimicrobial peptides
APIs	Active pharmaceutical ingredients
ARDS	Acute respiratory distress syndrome
cAMP	Cyclic adenosine monophosphate
cfDNA	Cell-free DNA
Co-Q10	Coenzyme Q10
DAMPs	Damage-associated molecular patterns
DNA	Deoxyribonucleic acid
DNase I	Deoxyribonuclease I
DPI	Diphenyleneiodonium
FLCs	Free light chains
GCF	Gingival crevicular fluid
GSDMD	Gasdermin D
H3Cit	Citrullinated histone 3
HMGB1	High mobility group box 1
IL	Interleukine
LDD	Local drug delivery
LXA4	Lipoxin A4
LPS	Lipopolysaccharide
MMP	Matrix metalloproteinase
MPO	Myeloperoxidase
NAC	N-acetyl cysteine
NADPH	Nicotinamide adenine dinucleotide phosphate
NCF2	Neutrophil cytosolic factor 2
NE	Neutrophil elastase
NETs	Neutrophil extracellular traps
Nexinhibs	Neutrophil-specific exocytosis inhibitors
NF-κB	Nuclear factor-kappa B
NO	Nitric oxide
NOX	Nicotinamide adenine dinucleotide phosphate-oxidase
PAD4	Peptidyl arginine deiminase-4
PAMPs	Pathogen-associated molecular patterns
PD	Periodontitis
*P. gingivalis*	*Porphyromonas gingivalis*
*P. intermedia*	*Prevotella intermedia*
PKC	Protein kinase C
PMNs	Polymorphonuclear neutrophils
PPAD	Peptidyl arginine deiminase
PR3	Proteinase-3
PRR	Pattern recognition receptors
PUFAs	Ω-3 polyunsaturated acids
RA	Rheumatoid arthrosis
RAGEs	Receptors for advanced glycation end-products
Rgp	Gingipain R
RNS	Reactive nitrogen species
ROS	Reactive oxygen species
RvTs	T-series resolvins
SIRL-1	Signal inhibitory receptor on leukocytes-1
SLE	Systemic lupus erythematosus
SNARE	Soluble N-ethylmaleimide-sensitive factor attachment protein receptor
*T. forsythia*	*Tannerella forsythia*
TLR	Toll-like receptor
TNF-α	Tumor necrosis factor-α
